# Predators Exacerbate Competitive Interactions and Dominance Hierarchies between Two Coral Reef Fishes

**DOI:** 10.1371/journal.pone.0151778

**Published:** 2016-03-18

**Authors:** April Hall, Michael Kingsford

**Affiliations:** College of Marine and Environmental Science and Centre of Excellence in Coral Reef Studies, James Cook University, Townsville, QLD, Australia 4811; Evolutionary Biology Centre (EBC), Uppsala University, SWEDEN

## Abstract

Predation and competition are critical processes influencing the ecology of organisms, and can play an integral role in shaping coral reef fish communities. This study compared the relative and interacting effects of competition and predation on two competing species of coral reef fish, *Pomacentrus amboinensis* and *P*. *moluccensis* (Pomacentridae), using a multifactorial experiment. Fish were subjected to the sight and smell of a known predator (*Pseudochromis fuscus*), the presence of the heterospecific competitor (i.e., *P*. *amboinensis* vs. *P*. *moluccensis*), or a combination of the two for a period of 19 days. The sub-lethal effects of predator/competitor treatments were compared with controls; a combination of otolith microstructure analysis and observations were used to determine otolith growth patterns and behaviour. We predicted that the stress of competition and/or predation would result in strong sub-lethal impacts, and act synergistically on growth and behavioural patterns. We found strong evidence to support this prediction, but only for *P*. *amboinensis*, which suffered reductions in growth in both predator and competitor treatments, with the largest reductions occurring when subjected to both predation and competition concurrently. There was strong evidence of asymmetrical competition between the two damselfish species, with *P*. *moluccensis* as the dominant competitor, displaying strong aggressive behaviour towards *P*. *amboinensis*. Growth reductions for *P*. *amboinensis* in predator/competitor treatments appeared to come about primarily due to increases in shelter seeking behaviour, which significantly reduced the foraging rates of individuals compared with controls. These data highlight the importance of predator/competitor synergisms in influencing key behaviours and demographic parameters for juvenile coral reef fishes.

## Introduction

Predators play a crucial role in both marine and terrestrial environments, and patterns of predation can be a strong determinant of community structure. Predators exert top-down control on lower trophic levels through their interactions with prey, which may be important in shaping communities through time [1–3]. Predators influence prey dynamics primarily through direct mortality, however the presence of a predator may alter the demographic and behavioural traits of prey, resulting in a variety of sub-lethal effects. On coral reefs, predatory fishes can play a strong role in regulating prey communities, and key demographic traits such as growth and colour patterns, size and age structures, condition and reproductive output of prey species may be influenced by local predator densities [4–9]. Demographic traits are often the result of predator-induced behavioural modifications, which can reduce the availability of energy for growth and reproduction [10–12]. When predator biomass is high, prey may need to allocate more energy to predator avoidance, and may also reduce their energy intake by feeding less, or consuming less nutritious prey [12]. Such “risk effects” have been demonstrated in several studies, which have documented the behavioural and demographic response of juvenile reef fishes to predators [1, 4, 5, 13–18].

Competition can also shape communities, as individuals compete for finite resources such as food, mates, or shelter space [19]. In a competitive interaction, dominant individuals may actively restrict subordinates from accessing resources using aggressive displays, and this process can regulate populations by limiting the capacity of subordinate individuals to grow and reproduce [20]. This can result in asymmetrical competition, whereby the subordinate competitor is negatively impacted whilst the dominant competitor is unaffected [21]. In many ecological systems, the processes of competition and predation are tightly linked, and often interact to determine mortality rates and population densities of lower trophic level prey species [22–25]. Interactions between competition and predation may vary amongst systems, depending on a number of factors such as the limiting resource, the magnitude of the predator threat, and the social dynamics of each species [24, 26]. The combined effects of competition and predation on prey mortality may be inhibitory (where the combined effects are less than the sum of individual effects), additive (where the combined effects equal the sum of individual effects) or synergistic (where the combined effects are greater than the sum of individual effects; [27].

Inhibitory interactions can occur when predators mediate competition amongst prey, by removing individuals and preventing complete dominance of one individual or species [25, 26]. Such interactions have commonly been observed in intertidal systems, where competition occurs primarily for optimal position within the intertidal zone [25, 28, 29]. Alternatively, predation may increase the intensity of competitive interactions, resulting in additive or synergistic effects on key life history traits. This can occur when species compete for access to predator-free shelter sites, or when competitive interactions increase the vulnerability of prey to predators by impacting growth rates [30]. Such effects are commonly observed for teleost fishes, where size-selective predation is often observed, and growth and condition are critical factors which influence survivorship of prey species [31, 32].

In coral reef fish communities, the combined processes of predation and competition are critical to population regulation, and juveniles may be particularly vulnerable due to their bipartite life cycle [1]. As a naïve individual transitions from its pelagic larval stage to its demersal reef-associated stage, it is exposed to a suite of novel predators, with mortality levels reaching up to 90% within the first 48 hours of settlement [33, 34]. Surviving fish must then compete for critical resources such as food and shelter space, and competition amongst conspecifics and heterospecifics can be intense, with dominance hierarchies forming quickly after settlement [35, 36]. Competitively dominant individuals may increase the mortality of subordinates by restricting access to key shelter sites, thereby increasing predation risk [22, 23, 37]. Competitively dominant group members may prevent subordinates from accessing food resources or foraging positions, which can have major consequences for growth rates [38, 39]. Given the importance of a size advantage in avoiding predation, variations in growth rates can have major consequences for survivorship [31, 32, 40]. This early post-settlement stage is a critical time for development for small coral reef fishes as reductions in key demographic parameters such as growth rates can influence life time survivorship and reproductive output [41].

Since growth rates can be a key determinant of prey populations at the community level, understanding the factors influencing demographic processes at these early life stages is a critical component of coral reef ecology. The development of otolith analysis techniques has contributed considerably to this field, since analysis of otolith increment widths can provide a record of the growth of individuals over time [42]. Otoliths are calcium carbonate structures which accumulate daily growth rings that are related to the diel physiological cycles of fishes [42, 43]. The daily growth (increment width) of otoliths can be used as a proxy for somatic growth, and can provide a chronological record of past growth, which can then be related to natural conditions and/ or experimental treatments. This is a commonly used approach in demographic studies, and the relationship between daily increment widths and somatic growth has been validated for several tropical fish species including *P*. *amboinensis* [44–47]. The objective of this study was to use growth data derived from otoliths in conjunction with behavioural data to investigate the importance of predation and inter-specific competition for two species of competing damselfish; *Pomacentrus amboinensis* (ambon damsel) and *P*. *moluccensis* (lemon damsel). We focused on growth rates and behaviour during the juvenile life stage. Our initial predictions were as follows: (1) the presence of a predator or, (2) a heterospecific competitor would result in decreased growth, and changes in foraging behaviour and general activity of juvenile prey and, (3) the presence of both a predator and heterospecific competitor would exacerbate the aforementioned sub-lethal effects, resulting in synergistic impacts on otolith growth. To address these predictions, this study aimed to:

1. Investigate the relative and interacting effects of predator threat and interspecific competition on the otolith growth of *P*. *amboinensis* and *P*. *moluccensis;*
2. Investigate competitive interactions between *P*. *amboinensis* and *P*. *moluccensis* and examine patterns of interspecific aggression and dominance hierarchies;
3. Examine the combined effects of predation and interspecific competition on otolith growth, foraging and sheltering behaviour, and competitive interactions for *P*. *amboinensis* and *P*. *moluccensis*.

## Materials and Methods

Three species of fish were used in the experiment: juvenile *Pomacentrus amboinensis* (ambon damsel; prey species one), juvenile *P*. *moluccensis* (lemon damsel; prey species two), and adult *Pseudochromis fuscus* (yellow dottyback; predator). These three species have been used extensively in behaviour-focused predator-prey experiments, where juveniles of both damselfish species have been reported to exhibit a behavioural response to the sight and smell of *P*. *fuscus*, the latter being a voracious predator of juvenile damselfishes [15, 48–50]. *P*. *amboinensis* and *P*. *moluccensis* are both small, common, site-attached damselfishes (Pomacentridae) which often co-inhabit coral patch reefs, and may compete for key resources such as food and shelter, particularly in the juvenile stages [35]. Both species preferentially recruit to live coral, however *P*. *amboinensis* may inhabit dead coral rubble habitats as adults [51]. *P*. *fuscus* (Pseudochromidae) is a small piscivorous predator, and may inhabit patch reefs alongside them, preying opportunistically on new recruits and juveniles [49]. *P*. *fuscus* has been used extensively in experimental trails, where predation on both *P*. *amboinensis* and *P*. *moluccensis* has been commonly observed in aquarium environments [32, 40].

This study was conducted from December 2013 to January 2014 at Lizard Island Research Station, at the northern end of the Great Barrier Reef, Australia. All fish were caught off shallow patch reefs at multiple sites around Lizard Island using diluted clove oil and hand nets. Small pieces (≈ 5 x 5 x 5cm) of live *Pocillopora damicornis* (cauliflower coral) were obtained from similar sites using chisels, to use for shelters in the experiment tanks. *P*. *damicornis* is an abundant coral species at sites around Lizard Island, and commonly inhabited by both damselfish species. Coral pieces were carefully selected to be of similar size and structural complexity. After collection, all fishes/ corals were transported immediately back to the research station, and held in flow through aquaria for at least four days before being used in the experiment, to allow them to acclimate to the experimental conditions. All *P*. *amboinensis* and *P*. *moluccensis* measured 13–17mm SL (average = 14.8mm), and *P*. *fuscus* measured 75–90mm at the commencement of the experiment. As both damselfish species were captured off the reef and had likely been there for 20–30 days based on their size, we assumed that they would be familiar with the sight and scent of reef predators such as *P*. *fuscus*. *P*. *amboinensis* and *P*. *moluccensis* were fed twice daily with 5 ml (per fish) of concentrated *Artemia (≈* 600 *Artemia* per mL) in all experimental treatments, and *P*. *fuscus* individuals were fed two damselfish recruits morning and night throughout the experimental and holding period. This is an approximate representation of what *P*. *fuscus* would consume in the wild [49], ensuring that the predator stimulus was realistic. Where possible, *P*. *fuscus* were fed conspecifics (according to each treatment type), with the skin lacerated to ensure that the water was scented with chemical alarm cues [52]

This study was carried out in strict accordance with the National Health and Medical Research Council, *Australian Code for the Care and Use of Animals for Scientific Purposes*, and in compliance with the *Queensland Animal Care and Protection Act*, *2001* and James Cook University (JCU) guidelines. The Animal Ethics Committee at JCU approved the protocol used in this study (approval # A1808). All research was carried out within the Great Barrier Reef Marine Park by permit from the Great Barrier Reef Marine Parks Authority (permit # G12/35131.1). This study did not involve endangered or protected species.

### 1. Experimental setup

The aquarium layout was designed so that prey species could both see and smell the predator, but could not be accessed by it (S1 Fig). To achieve this, small experiment tanks (LxWxH = 20cm x 10cm x 10cm) were placed inside larger, opaque holding tanks (LxWxH = 43cm x 32cm x 31cm) which received flow-through ambient seawater. The smaller, experiment tanks were made of transparent plastic and contained vents, which allowed water to flow freely between the two tanks (S1 Fig). The experimental tanks housed the prey species, along with their coral shelter, whilst the holding tank either contained the predator, or was empty, according to the treatment type. Competition treatments were created by adding either a conspecific or heterospecific to the experimental tanks (as outlined below). A feeding tube made from soft tubing was attached to the top of each experimental tank, which allowed the *Artemia* to be injected into the tank from a distance, so that the experimenter was not seen, and fish were not disturbed during behavioural trials. The feeding tube was used for the duration of the experiment. The amount of *Artemia* (5 ml per fish) was kept constant across treatments, and was added to each tank slowly, at a constant rate, to allow all fish equal access to food resources.

### 2. Experimental design

A fully orthogonal two-factor design was used to test experimental treatments for each species. Factors were predator presence (two levels) and competitor presence (three levels; Table 1). The orthogonal design comprised of 10 total treatments, with six replicate experiment tanks per treatment (Table 1), and the experiment was run for a total of 19 days. This time period is biologically relevant, given the importance of growth during the first few months post-settlement, and the strong link between size and mortality during this time [31{Figueira, 2008 #569, 32]. We hypothesized that if experimental treatments had a strong impact on growth, then variations in growth trajectories would be detectable within this time period. Treatments were randomised amongst tanks, and the allocation of captured fish from different patch reefs was randomized so that each treatment contained a random sample of fish. This method ensured that local variations in predator and/ or competitor interactions did not confound experimental treatments. Details of each treatment are outlined below (PA = *P*. *amboinensis* and PM = *P*. *moluccensis*):

**Table 1:** Sampling design of experiment, with treatment names as they are referred to throughout the text. PA = *Pomacentrus amboinensis*, PM = *Pomacentrus moluccensis*. Numbers refer to fish per replicate tank (e.g. PA2 = 2 PA per tank), PA: PM indicates one individual of each species per replicate tank.

	Fish per replicate experiment tank			Purpose of treatment		
Treatment name	Predator	PA	PM	Controls for	Tests for	Replicates
No predator PA1	No	1	0	Predator	-	6
No predator PA2	No	2	0	Predator and interspecific competitor	-	6
No predator PM1	No	0	1	Predator	-	6
No predator PM2	No	0	2	Predator and interspecific competitor	-	6
No predator PA:PM	No	1	1	Predator	Interspecific competitor	6
Predator PA1	Yes	1	0	-	Predator	6
Predator PA2	Yes	2	0	Interspecific competitor	Predator	6
Predator PM1	Yes	0	1	-	Predator	6
Predator PM2	Yes	0	2	Interspecific competitor	Predator	6
Predator PA:PM	Yes	1	1	-	Predator and interspecific competitor	6
					**Total**	**60**

#### i) Competition treatments

Interspecific competition was tested using three competition treatments for each species as follows: 1) No competitor (PA1 and PM1); 2) paired conspecifics (PA2 and PM2); and; 3) paired heterospecifics (PA1:PM1). The no competitor treatments contained a single fish and were used to test for predator effects only. Paired conspecific treatments were used as a control for density, so that equal densities occurred between paired conspecific and paired heterospecifics treatments. This allowed the effects of the heterospecific competitor to be separated from any effects that may be attributable to changes in density, and not to the identity of the competitor *per se* (see Table 1). A*ll tan*ks contained a single *P*. *damicornis* fragment. *Pairs were siz*e matched to 0.1 mm (SL) to remove any effect of a size-advantage on competitive outcomes.

#### ii) Predator treatments

We used two predator treatments (predator present and absent; Table 1). A single *P*. *fuscus* was added to the holding tank for each of the predator present treatments, such that it could swim freely around the smaller experimental tank. *P*. *fuscus* were rotated amongst predator treatment tanks every four days, to remove potential bias associated with individual traits of any predator.

### 3. Behavioural observations and dominance hierarchies

#### i) Observation protocol

To evaluate the influence of predators and competitors on prey, behavioural observations were undertaken on day 17 of the experiment. The behaviour of fish in each experimental tank was recorded using GoPro cameras, placed inside the holding tank and facing the experimental tank. Behaviour was recorded for a total of seven minutes, including an initial one minute acclimation period, to allow fish to settle from any disturbance caused by adding the camera to the tank. After this initial minute, recording continued for a further three minutes before food was discretely added to the tank using the feeding tube; recording continued for another three minutes post-feeding. Each recorded video was then watched by the same observer, and the: foraging rate, and activity of the fish (swimming (without foraging), sheltering, or foraging (either whilst swimming or stationary)) was recorded every 10 seconds. The foraging rate was determined by recording the number of feeding strikes (successful or not) towards *Artemia* in the water column, and converting this into a foraging rate (per minute). Fish were recorded as sheltering if they were stationary at <1cm from their coral shelters. An average was taken from the 36 time points during the recording to calculate the percentage time spent in each activity for each fish. Agonistic/ aggressive interactions (defined as a nip or chase) were recorded per minute in the competition treatments. Foraging behaviour was only recorded in the post-feeding time period; all other behaviours (swimming, sheltering and agonistic interactions) were recorded over the full six minute period.

#### ii) Dominance hierarchies

We identified dominant and subordinate individuals through behavioural observations, and used associated growth data to test for asymmetrical competition. For paired competitors, each individual was defined as either dominant or subordinate by quantifying patterns of aggression. *Dominant* individuals were defined as such if they initiated the majority (>90%) of aggressive interactions (i.e. chases or nips) during competitive interactions. *Subordinate* individuals were defined as such if they initiated few (<10%) or no aggressive interactions, and exhibited avoidance behaviour (i.e. retreating from/ avoiding the dominant individual) in response to aggression. Dominance hierarchies from behavioural data were compared to growth data to detect evidence of asymmetrical competition. *Asymmetrical competition* was defined as a reduction in a key trait (i.e. growth) for a subordinate species, whilst the dominant species remained unaffected. Asymmetrical competition was examined by comparison of growth trajectories for each species when in the presence/ absence of a heterospecific competitor.

### 4. Growth effects: otolith increment width analysis

We used daily increment widths from the otoliths of *P*. *amboinensis* and *P*. *moluccensis* to measure the growth of otoliths as a proxy for the growth of individuals during the experiment. The starting point of the experiment was determined by counting back 19 daily increments within the otolith. Data from days 1–19 of the experiment were used to calculate cumulative increment widths. We used otolith growth rather than direct measurements of somatic growth, since otolith increments give a daily representation of the biological response of the fish to experimental treatments without subjecting fish to stress from daily measurements of somatic growth, which may influence experimental outcomes. All references to growth throughout refer to otolith growth, obtained from measurement of daily increment widths. At the conclusion of the experiment, fish were sacrificed using an ice water bath, measured (SL) to the nearest 0.1mm and their otoliths extracted. Sagittal otoliths were removed, cleaned and ground to obtain a thin transverse section through the primordium. Samples were coded so their identity was unknown when measuring increment widths. Otolith sections were then polished until the daily rings were clear, and the daily increment widths (i.e. distance between rings) corresponding to the experimental period were measured using a calibrated computer program.

### 5. Data treatment and statistical analyses

The cumulative daily otolith increment widths of each individual were calculated, and repeated measures analysis of variance (RMANOVA) were used to compare growth trajectories over time in the predator and competitor treatments. Single factor RMANOVA was used to test for the effects of the predator or competitor, and two-factor RMANOVA was used to compare growth trajectories according to predator and competitor treatments. To test for the effects of interspecific competition, growth trajectories for paired conspecific treatments (i.e. PA2 or PM2; control) were compared against paired heterospecifics (PA1:PM1). To determine the magnitude of effects of competition, predation, or a combination of the two on otolith growth, effect sizes were calculated for each treatment. Cohen’s d values [53] were calculated as a measure of effect sizes, by comparing the mean of each treatment with its appropriate control as follows:

$$d\;=\;\frac{M_{group1}-\;M_{group2}}{{SD}_{pooled}}$$

Where: M = mean

   Group 1 = experimental treatment (e.g. PA1:PM1)

   Group 2 = appropriate control (e.g. PA2)

$${SD}_{pooled}\;=\;\sqrt{({{SD}^{2}}_{group1}+\;{{SD}^{2}}_{group2\;})/2}$$

Cumulative increment widths from the final day of the experiment were used to calculate Cohen’s d for *P*. *amboinensis* and *P*. *moluccensis*, which gave an estimate of the consequences of each treatment on otolith growth at the conclusion of the experiment. Effect sizes were used to assess the relative importance of predation alone, competition alone, and both competition and predation. Comparisons of effect sizes amongst treatments were used to determine whether the combined effects of predation and competition were inhibitory, additive or synergistic.

Two sample t tests were used to compare the behaviour of *P*. *amboinensis* and *P*. *moluccensis* amongst experimental treatments. T tests were used to compare differences in foraging rates, and activity patterns between predator treatments (PA1 and PM1), between species within competition and predator treatments (PA1:PM1), and to compare aggressive interactions between species (PA1:PM1). Linear regression was used to examine the relationship between foraging rates and competitive behaviour (aggressive interactions initiated and avoidance behaviour) for both species. Data were pooled between species for the regression analysis to determine the overall consequence of the competitive behaviours on foraging rates. Assumptions of normality and homogeneity of variance were tested using Cochran’s test, as well as visual examination of the distribution of the residuals; data were transformed when necessary. Multivariate tests (Pillai’s trace) were used for the within component of the RMANOVA tests because they are more robust to violations of the assumptions of RMANOVA.

## Results

### Growth

#### 1. Effects of predator only

**Fig 1:**
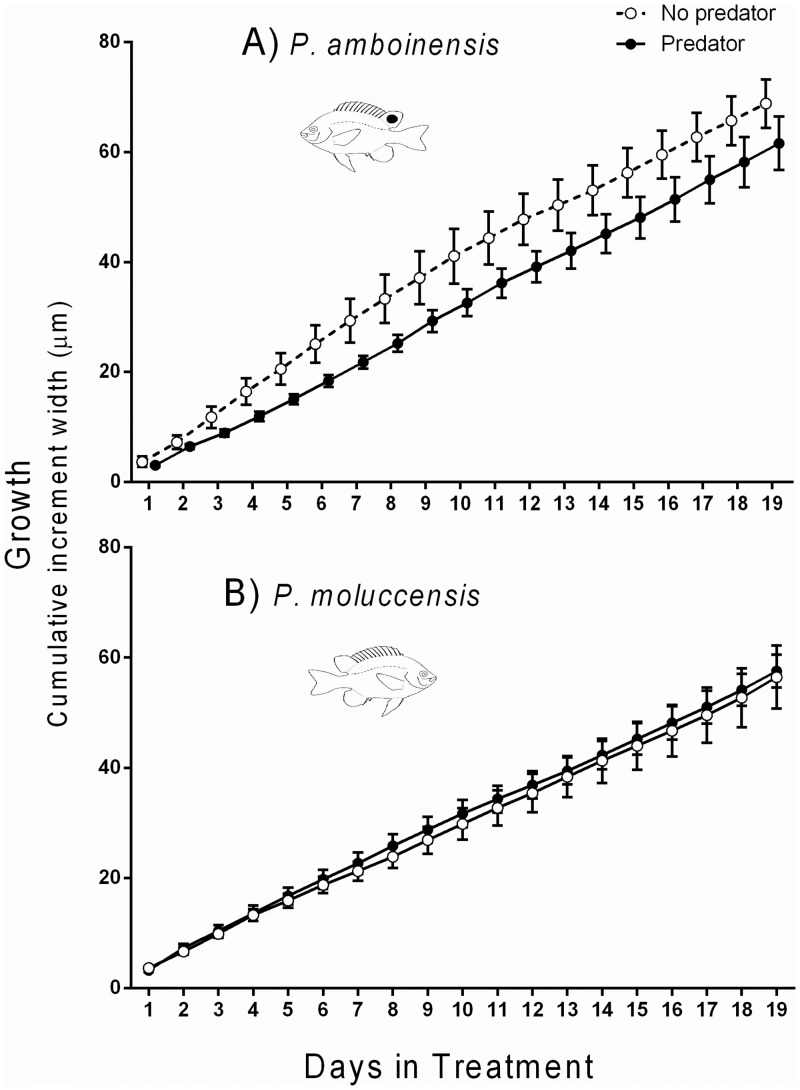
Growth (mean cumulative otolith increment width ±1 SE) of A) *P*. *amboinensis* and B) *P*. *moluccensis* during the experimental period according to predator treatment. All data are for single fish (PA1 and PM1) only with no competitor present.

The presence of the predator (*P*. *fuscus*) had a significant effect on the growth of *P*. *amboinensis* throughout the experiment (Fig 1A). Growth was reduced in the presence of the predator, and growth trajectories differed significantly between the predator treatments [RMANOVA (Day x predator treatment; F _(18,180)_ = 2.354, p = 0.002; Fig 1A]. Conversely, the presence of the predator had no detectable effect on the growth of *P*. *moluccensis*, and growth trajectories between predator treatments were similar over time [RMANOVA (Day x predator treatment) F _(18,180)_ = 0.080, p = 1.000; Fig 1B].

#### 2. Effects of competitor only

**Fig 2:**
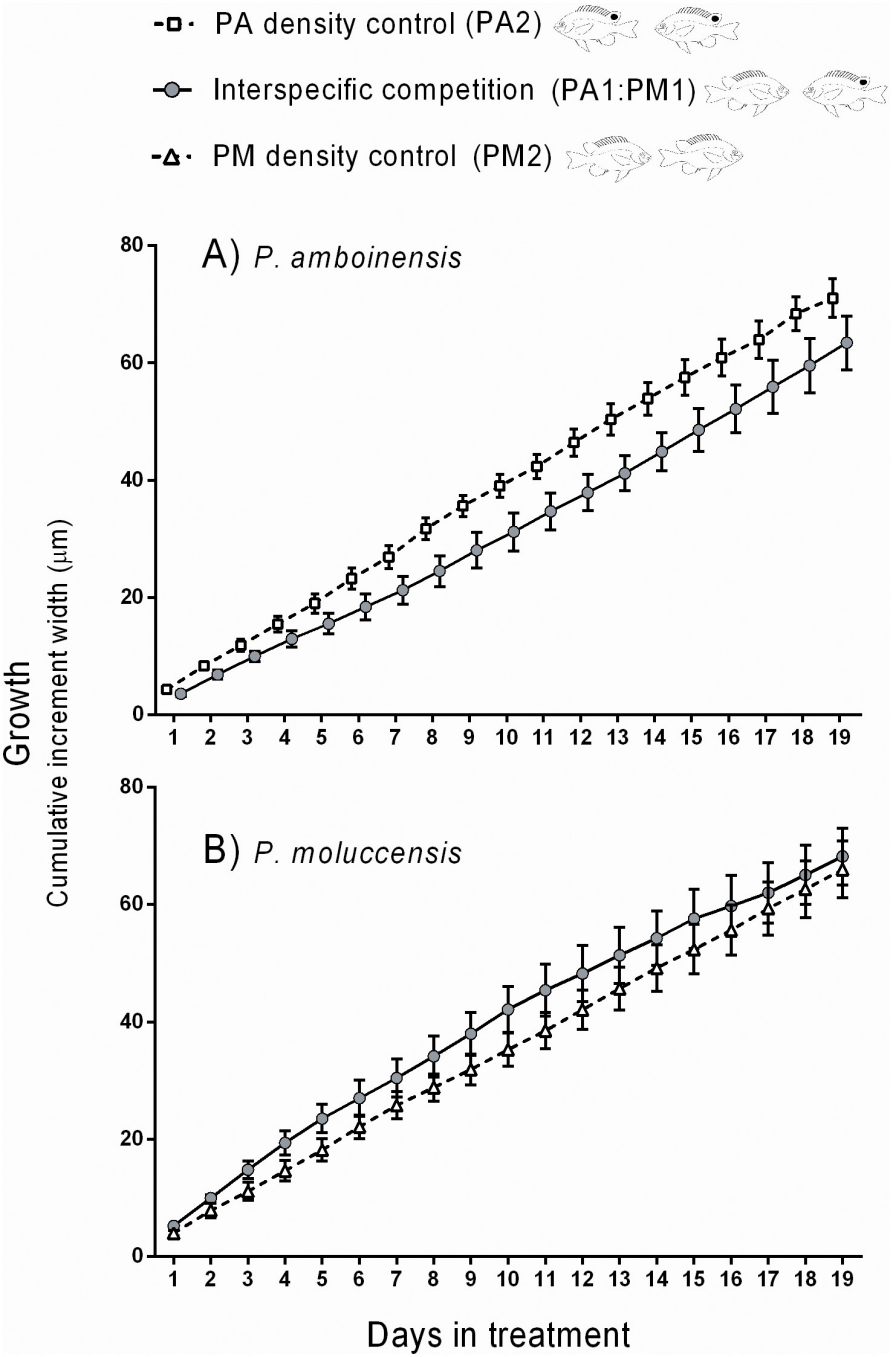
Growth (mean cumulative otolith increment width ±1 SE) of A) *P*. *amboinensis* and B) *P*. *moluccensis* during the experimental period according to competitor treatment, with no predator present. Paired conspecific treatments (PA2 and PM2) are controls for the paired heterospecific (interspecific competition) treatment (PA1:PM1). PA = *P*. *amboinensis*, PM = *P*. *moluccensis*.

The presence of the heterospecific competitor (*P*. *moluccensis*) had a significant negative effect on the growth of *P*. *amboinensis*. Growth trajectories were significantly lower in the paired heterospecific treatment (PA1:PM1), compared to the paired conspecific (PA2; control) treatment [(RMANOVA (Day x competitor treatment) F _(18,180)_ = 11.390, p<0.001; Fig 2A]. Conversely, growth of *P*. *moluccensis* was not negatively affected by the interspecific competition treatment, and growth trajectories in the paired heterospecific (PA1: PM1) treatment were similar to the paired conspecific (PM2; control) treatments [RMANOVA (Day x competitor treatment) F _(18,180)_ = 8.390, p<0.925; Fig 2B].

#### 3. Interactive effects of predator and competitor

**Fig 3:**
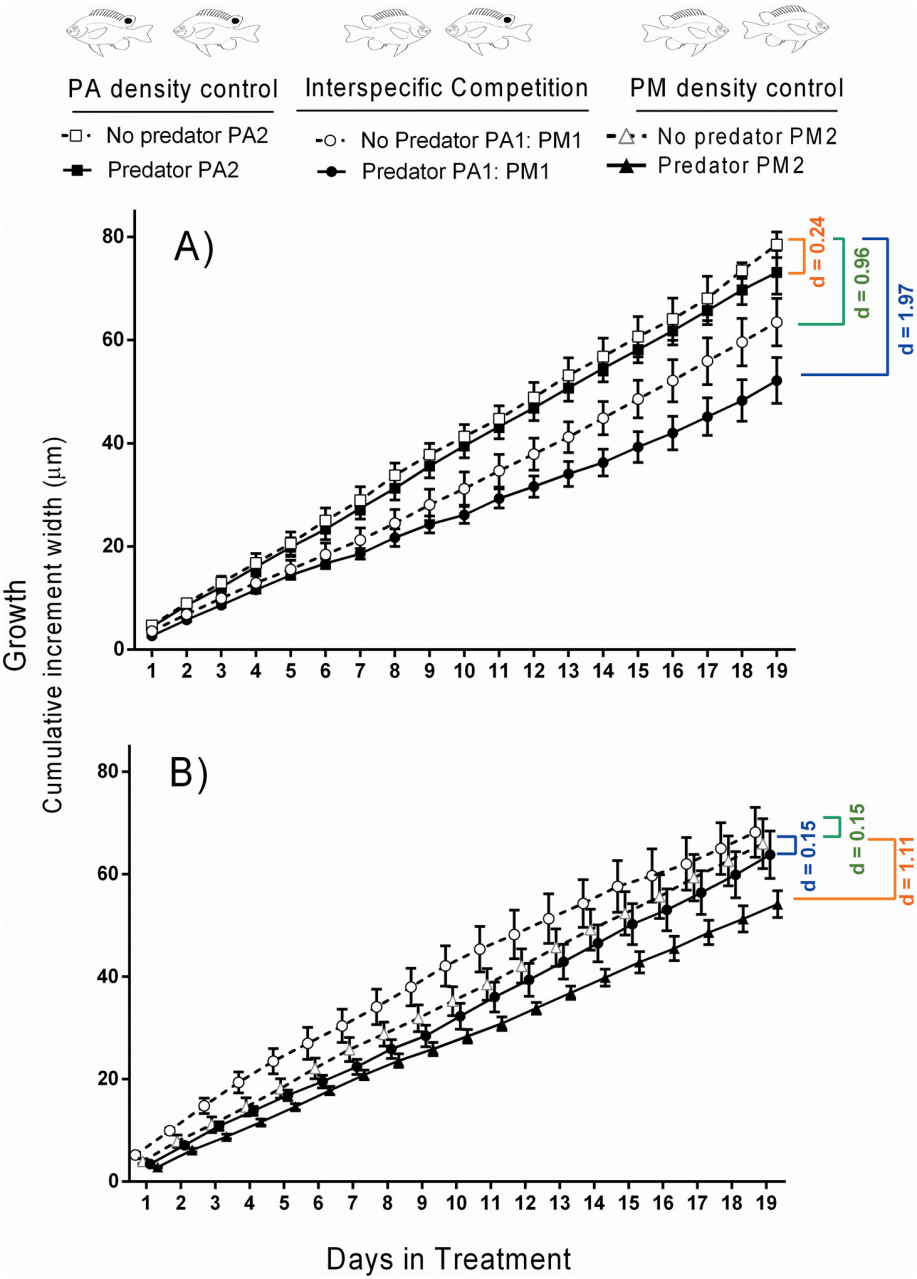
Growth (mean cumulative otolith increment width ±1 SE) of A) *P*. *amboinensis* and B) *P*. *moluccensis* during the experimental period according to predator and competitor treatments. Paired conspecific treatments (PA2 and PM2) are controls for the paired heterospecific (interspecific competition) treatment (PA1:PM1). PA = *P*. *amboinensis*, PM = *P*. *moluccensis*. Colours correspond to effect sizes (Cohen’s d values) as follows: orange = predator effects only, green = competitor effects only, blue = both predator and competitor effects. Effect sizes were calculated from day 19 cumulative growth data.

The growth of *P*. *amboinensis* was affected by interactions between the presence of the predator and interspecific competitor (*P*. *moluccensis*). Comparison of effect sizes (Cohen’s d) showed that interspecific competition (d = 0.96) had a greater effect on the growth of *P*. *amboinensis* than predation (d = 0.24; Fig 3A). Growth was lower in the presence of the predator in both competition treatments, however the magnitude of difference varied between competition treatments resulting in a significant interaction between predation and competition [RMANOVA (predator treatment x competitor treatment); F _(2, 30)_ = 5.895 p = 0.007]. The combined effects of interspecific competition and predation (d = 1.97) were greater than the additive effects of predation and competition alone (0.24+0.96 = 1.20), indicating a synergistic effect of competition and predation for *P*. *amboinensis* (Fig 3A). These effects increased over time, and growth trajectories between predator treatments in the interspecific competition treatment became more disparate throughout the experimental period [RMANOVA (day x predator treatment x competition treatment); Pillai’s trace _(36, 28)_ = 2.689, p = 0.004].

In contrast, *P*. *moluccensis* did not experience significant reductions in growth due to interactions between the heterospecific competitor and predator (Fig 3B). Although there were significant differences in the growth trajectories amongst competition treatments through time [RMANOVA (day x competition treatment); Pillai’s trace _(36, 28)_ = 2.552, p = 0.006], growth was actually greater in the heterospecific competition treatments (PA1:PM1) compared to the paired conspecific treatments (PM2; control; Fig 3B). This trend was stronger in treatments where a predator was also present, and the effect size of competition in the absence of a predator was relatively small (d = 015). In the density control treatments (PM2), growth was lower in the presence of the predator (d = 1.11), but the overall effect of the predator on growth trajectories was not significant [RMANOVA (day x predator treatment); Pillai’s trace _(18, 13)_ = 2.223, p = 0.074]. The combined effects of interspecific competition and predation (d = 0.15) were less than the additive effects of predation and competition (d = 0.15+1.11 = 1.26), as well as the individual effect of predation (d = 1.11), indicating an inhibitory effect of predation and interspecific competition combined (Fig 3B).

### Behaviour

#### 1. Behavioural response to predator only

**Fig 4:**
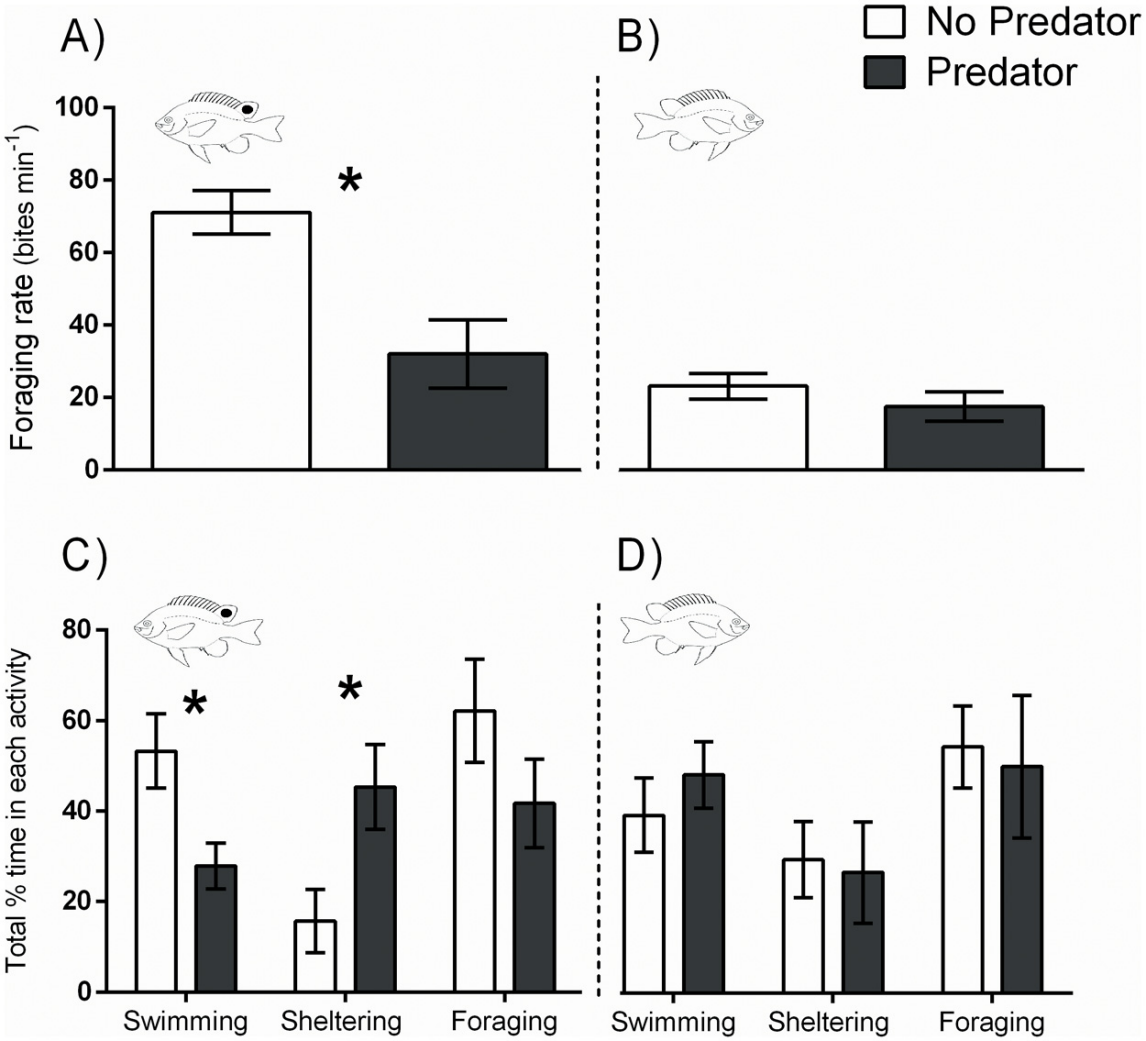
Foraging rates of *P*. *amboinensis* (A) and *P*. *moluccensis* (B), and activity patterns of *P*. *amboinensis* (C) and *P*. *moluccensis* (D), between predator treatments. All data are for single fish (PA1 and PM1) only with no competitor present. Asterisks indicate significant differences between predator treatments for each species (t-tests); all bars show means ±1 SE.

Both *P*. *amboinensis* and *P*. *moluccensis* changed their behaviour in the presence of the predator; however, behavioural changes were much stronger and more consistent for *P*. *amboinensis* (Fig 4). *P*. *amboinensis* significantly reduced their foraging rates in the presence of the predator, and mean foraging rates were reduced by 48% [(t-test,t_10_ = 3.486, p = 0.0059; Fig 4A]. There was a similar trend for *P*. *moluccensis*, which exhibited a 29% reduction in foraging rates, however this difference was not significant [(t-test,t_10_ = 1.045, p = 0.3266; Fig 4B].

*P*. *amboinensis* showed strong changes to overall activity patterns according to the presence/ absence of the predator (Fig 4C). *P*. *amboinensis* spent around half the amount of time swimming (t-test: t_10_ = 2.628, p = 0.0252) and almost tripled the time spent sheltering (t-test: t_10_ = 2.540, p = 0.0294) when the predator was present. There was also a trend for *P*. *amboinensis* to spend more time foraging when the predator was absent, however this was not significant (t-test: t_10_ = 1.368, p = 0.2013). In contrast, *P*. *moluccensis* did not show any consistent changes to overall activity patterns according to the predator presence, and the percentage time spent swimming, sheltering and foraging was similar between predator treatments (Fig 4D).

#### 2. Behavioural response to predator and competitor treatments

**Fig 5:**
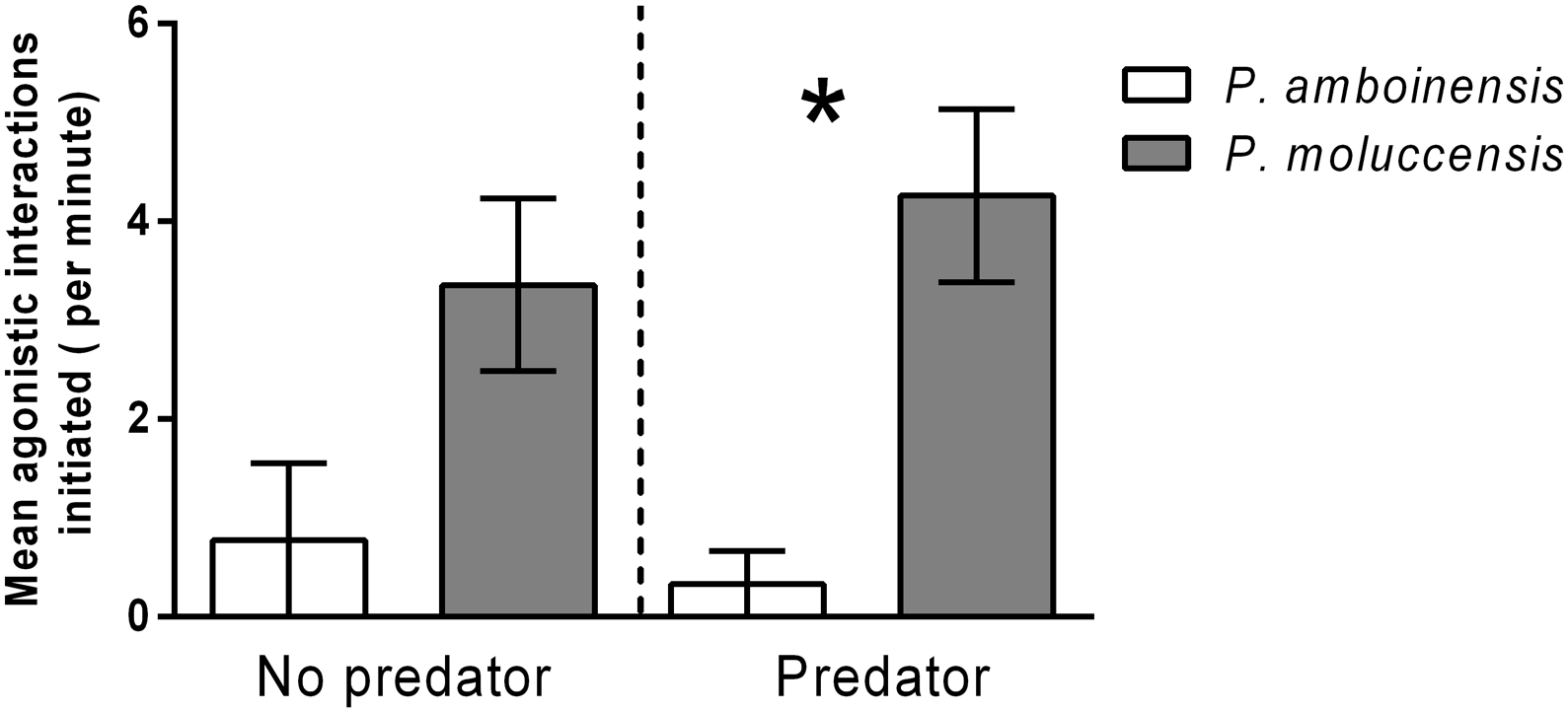
Mean (±1 SE) agonistic interactions (i.e. chases) initiated by *P*. *amboinensis* and *P*. *moluccensis* in the interspecific competition treatment (PA1:PM1), separated by predator absence or presence. Asterisks indicate significant differences between species within each predator treatment (t-tests).

*P*. *moluccensis* was the dominant competitor in 10 out of 12 (83%) of competitive pairs, and initiated the majority of agonistic interactions during behavioural observations. The presence of the predator strengthened dominance hierarchies; whilst there was a trend for *P*. *moluccensis* to initiate more agonistic interactions than *P*. *amboinensis* in both predator treatments, this was only significant in the presence of the predator (t- test t_10_ = 4.197, p = 0.0018; Fig 5). Agonistic interactions generally involved a nip or a chase by the dominant fish (usually *P*. *moluccensis*), with avoidance behaviour exhibited by the subordinate fish (usually *P*. *amboinensis*).

**Fig 6:**
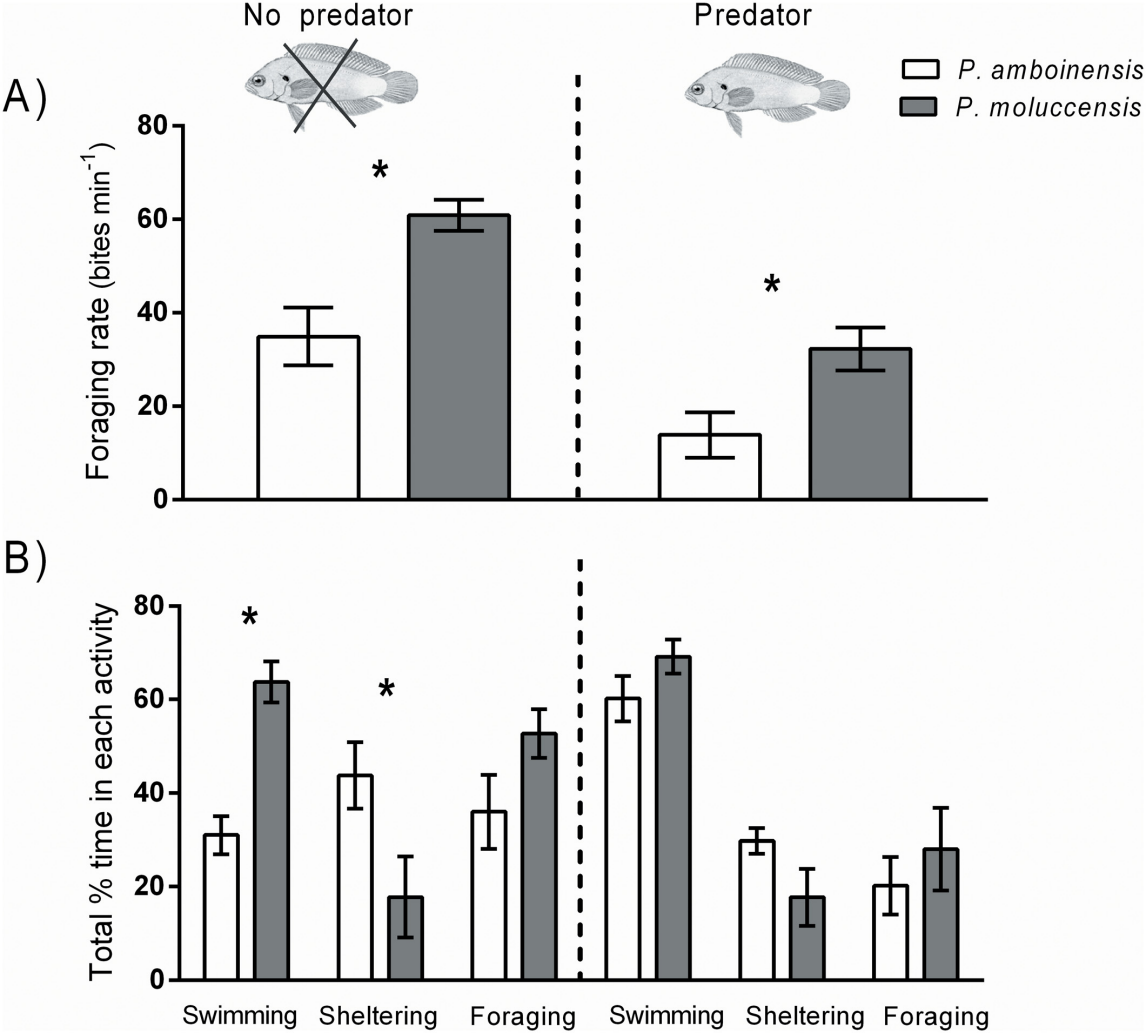
A) Foraging rates, and B) activity patterns of *P*. *amboinensis* and *P*. *moluccensis* in the interspecific competition treatments (PA1: PM1), separated by predator absence (left panels) or presence (right panels). Asterisks indicate significant differences between species within each predator treatment (t-tests); all bars show means ±1 SE.

**Fig 7:**
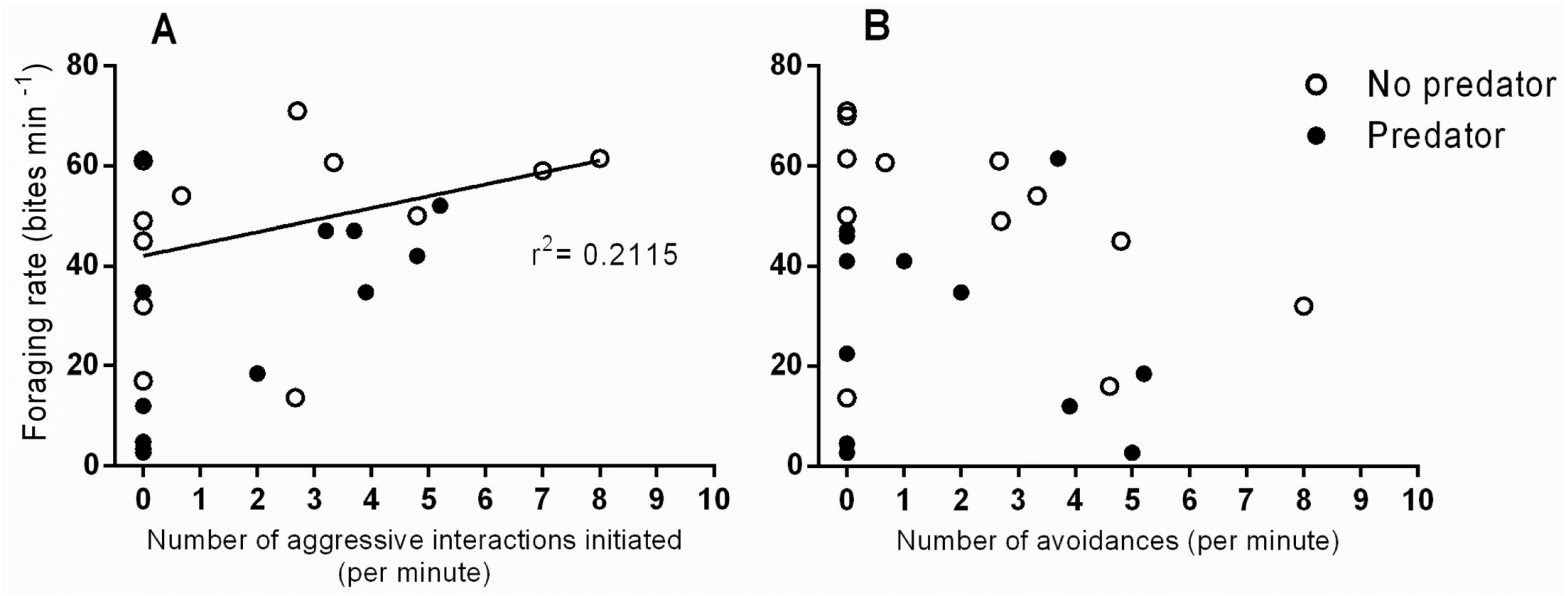
Relationship between foraging rates and A) the number of aggressive interactions (i.e. chases) initiated, and B) the number of avoidances (i.e. retreats) displayed for *P*. *amboinensis* and *P*. *moluccensis* (species pooled) in predator and no predator treatment. Line of fit on panel A represents that the slope is significantly different from zero.

*P*. *moluccensis* had a greater foraging rate compared to *P*. *amboinensis* when the predator was absent (t-test t_10_ = 3.710, p = 0.006; Fig 6A) as well as present (t- test t_10_ = 2.769, p = 0.0243; Fig 6A). The lower foraging rates for *P*. *amboinensis* were generally associated with competitive interactions, whereby *P*. *moluccensis* actively prevented *P*. *amboinensis* from accessing food through aggressive interactions (Fig 7). There was a significant positive relationship between foraging rates and the number of aggressive interactions (chases) initiated (test for slope (ANOVA); p = 0.0238; r^2^ = 0.2115), and a negative but non-significant relationship between foraging rates and the number of times a fish exhibited avoidance behaviour (test for slope (ANOVA); p = 0.292; r^2^ = 0.0503). Overall foraging rates were generally lower for both species in the predator present treatment.

Activity patterns differed between species, and *P*. *moluccensis* spent more time swimming, and less time sheltering compared to *P*. *amboinensis*. This trend occurred in both predator treatments, but was only significant when the predator was absent (t-test (swimming); t_10_ = 5.486, p = 0.0006, t-test (sheltering); t_10_ = 6.812, p = 0.0003 Fig 6B). There was a trend for *P*. *moluccensis* to spend more time foraging compared to *P*. *amboinensis*, however this was not significant in either predator treatment.

## Discussion

### Species comparisons: response to predator vs. competitor

Predation is a critical process influencing the distribution and abundance of reef fishes, and numerous experimental and observational studies have demonstrated the role of piscivorous fishes in influencing prey communities [1, 3, 5, 18, 54, 55]. We predicted that the presence of a commonly encountered predator (*P*. *fuscus)* would have considerable sub-lethal effects on both prey species, leading to reductions in growth and behavioural changes. We found support for this prediction, however there were marked differences in the response of *P*. *amboinensis* and *P*. *moluccensis* to the presence of the predator (*P*. *fuscus*). In the presence of the predator *P*. *amboinensis* displayed threat-reducing behaviours; foraging less and sheltering more, which resulted in significant growth reductions over the experimental period. In contrast, *P*. *moluccensis* showed relatively minor changes to behaviours in response to the predator, with no consequence on growth trajectories. Such variation in responses is surprising, given that *P*. *fuscus* is a voracious predator of juvenile damselfishes [49] and that both species have been shown to respond to changes in predator abundances on experimental patch reefs [6, 56, 57] and on natural reefs [3, 55]. Previous studies have shown that *P*. *moluccensis* can exhibit a behavioural response when presented with predator and/or conspecific chemical alarm cues [15, 50], however data from the present study suggests that this may not necessarily translate into growth reductions for this species on time scales of less than 20 days.

Competitive dominance can be an important factor determining group organisation for many species, however defining dominance can be problematic. For group living species, the size of an individual often determines their rank within a group, so relative size can be used as a reliable proxy for social rank [39]. In some studies, occupation of shelter sites has been used to infer dominance, since access to shelter sites can have a strong impact on survival [21, 35]. A key test of the importance of dominance hierarchies, however, is in the translation of behaviours to demographic outcomes. In this study, we used otolith data to unequivocally demonstrate the effects of predator and competitor treatments on growth trajectories. We controlled for size and focussed on the relationship between behavioural traits and growth outcomes to determine dominance hierarchies between *P*. *amboinensis* and *P*. *moluccensis*. Such comparisons revealed strong evidence of asymmetrical competition, with *P*. *moluccensis* as the dominant competitor. These outcomes contrast to previous studies, which have placed *P*. *amboinensis* as the dominant competitor due to their position lower down on patch reefs and closer to shelter sites [35, 58]. Direct comparisons of shelter use and foraging rates, however, were not considered in these studies. Although growth and mortality were measured, the effects of interspecific competition could not be separated from changes in density, as there was no intraspecific density control [35, 58].

Historically there has been much debate over the relative importance of competition for reef fishes, as well as the primary limiting resources which species or individuals may compete over [59–63]. Optimal foraging behaviour requires a trade-off between sheltering from predators and foraging, so both food and shelter can potentially be important in competitive interactions, and both scenarios have been observed on coral reefs [64]. Competition amongst or within coral reef fish species has been shown to occur over reef habitats [37, 59, 65], and dominant species or individuals may prevent subordinates from accessing key shelter holes [37]. Studies on gregarious reef fishes have also demonstrated strong competition for optimal foraging positions [38, 66, 67] and the quality of food consumed by an individual may depend on their social rank and physical position within feeding groups [38, 39]. In this study, there was a clear and direct link between competitive behaviour, foraging rates and growth trajectories, suggesting that competition for food resources was a strong driver of growth. Variation in the response of each species to the interspecific competition treatments was largely due to the establishment of clear dominance hierarchies arising from strong interspecific aggression by *P*. *moluccensis*. Given the direct positive relationship between aggression and foraging rates, it is highly likely that interference competition was the primary driver of the reductions in growth rates for *P*. *amboinensis*. In our experimental setup, the access to the food resource was controlled for and kept constant amongst treatments, however a comparison of competitive interactions at a range of resource levels would be required to completely rule out resource competition as an alternative mechanism.

### Interacting effects of competition and predation

A key outcome of this study was that the effects of competition were exacerbated by the presence of the predator. Our data supported the prediction that a combination of predator and competitor threat would have a synergistic effect on growth and behaviour for the two prey species. As the subordinate species, *P*. *amboinensis* suffered synergistic effects when exposed to both a predator and competitor, since there were greater reductions in growth, compared to either treatment alone, or the sum of both treatments. In contrast, *P*. *moluccensis*, as the dominant competitor, did not experience such reductions in growth trajectories, and tended to have a growth advantage in the interspecific competition treatments. The combination of predation and competition was therefore inhibitory for this species. This variation in response between the two species appeared to be tightly linked to the outcome of competition since such strong asymmetry in competitive outcomes was observed. These interactions highlight how important competitive dominance can be in gaining a growth advantage, and support the emerging notion that competition and predation interact as agents of mortality on coral reefs [23, 32, 68]. Although predation is the ultimate cause of mortality, competition over resources such as food or shelter may lead individuals to be more vulnerable to predators, and ultimately increase mortality rates for the subordinate species [37].

Growth trajectories during the early life stages for coral reef fishes can play a strong role in determining life-long survivorship, since predation risk can be strongly dependent on size [40]. Although this study focussed on a limited time period during the post-settlement stage, the observed reductions in growth could affect lifetime survival, as they may result in a smaller size-at-age and lower body mass [31, 40]. Since many coral reef predators are gape-limited, even modest reductions in growth can significantly influence predation risk during early life stages [69]. Given the importance of growth during this critical period, any variation in the response of species to stressors may play a key role in influencing reef dynamics. In this study, predation and competition acted synergistically to reduce the growth of *P*. *amboinensis*, whereas *P*. *moluccensis* was not similarly impacted, and these effects were inhibitory for this species. This suggests that *P*. *moluccensis* would have a significant advantage as a competitor in the predator-rich environment inhabited by these two species. Our data highlight and emphasize the complexity of interactions between competition and predation, and suggest that the response of an individual to these processes can depend on their status within competitive hierarchies.

### Growth vs. behavioural responses

Comparison of growth trajectories, coupled with behavioural observations in this study, facilitated a detailed understanding of how individuals reacted to predator/ competitor threat, and how this ultimately translated to changes in growth. We found variations in the degree to which growth and behaviour mirrored one another, depending on the species and the behaviour in question. The behavioural response of *P*. *amboinensis* to the predator, for example, closely mirrored the growth data; *P*. *amboinensis* exhibited risk averse behaviour, resulting in lower foraging rates when the predator was present, which translated into reduced growth. *P*. *moluccensis*, in contrast, only displayed minimal changes to behaviour, with no detectable growth effect. The more cautious approach taken by *P*. *amboinensis* with regards to predator avoidance may reduce the likelihood of mortality on patch reefs in the wild, however the reductions in growth associated with sheltering lower down in the water column may be a significant fitness consequence. In contrast, the behaviours exhibited by *P*. *moluccensis* allowed this species to dominate in competitive interactions, and therefore gain greater access to foraging opportunities, but would could increase the threat of predation. The combination of predation and competition resulted in variable outcomes in terms of how behaviours changed. Interestingly, the presence of the predator in competitor treatments exacerbated some behaviours, such as the prevalence of agonistic interactions, but mediated others, such as differences in the swimming and sheltering behaviour between species. These variations may have come about due to the inter-dependence of certain behaviours, and the pre-occupation of both species with competitive interactions when the predator was present. Regardless, the key result was a decrease in growth for the subordinate species when the predator was present.

### Implications

Understanding the role that predation and competition play in driving ecological communities may be particularly important in environments where human influences have modified these processes. On the Great Barrier Reef, predatory fishes such as groupers, snappers and emperors are heavily targeted by fisheries, resulting in severe predator depletion at heavily fished locations [3, 70]. This loss of higher trophic levels has resulted in increases in the densities of lower level prey taxa such as damselfishes, and overall changes in the composition of fish communities at both broad and local scales [3]. For lower level prey species, this constitutes a change in both predation patterns, as predators are lost, and competitive interactions, as densities of conspecific or heterospecific competitors increase correspondingly. In addition, the nature of competitive interactions may be influenced by human impacts such as degradation of coral reef habitats, which can influence the strength of competitive interactions and reduce the availability of shelter sites for prey to escape from predators [65, 71]. Given the potential impact of human activities on both predation and competitive interactions, an understanding of the relative importance of each process, as well as their potential interactions, will be of great utility when considering the outcomes of future perturbations. This study focussed on the early life history of prey fishes, however reductions in growth during this phase can influence survivorship, and may ultimately determine the reproductive output of species and influence community composition. This study has demonstrated the important sub-lethal effects of competition and predation on two common coral reef fishes, and our data highlight the need for further species-specific studies to elucidate the relative importance of these critical ecological processes for a range of species, in order to predict how coral reef fish assemblages may respond to future change.

## Supporting Information

S1 Fig Diagram of experimental setup, showing placement of the smaller experimental tank inside the larger flow-through holding tank. (TIF)
