# Supplementary figures and images for: Predators Exacerbate Competitive Interactions and Dominance Hierarchies between Two Coral Reef Fishes
Source: PLoS One. 2016 Mar 18;11(3):e0151778. doi: 10.1371/journal.pone.0151778 (PMC4798715; doi:10.1371/journal.pone.0151778)

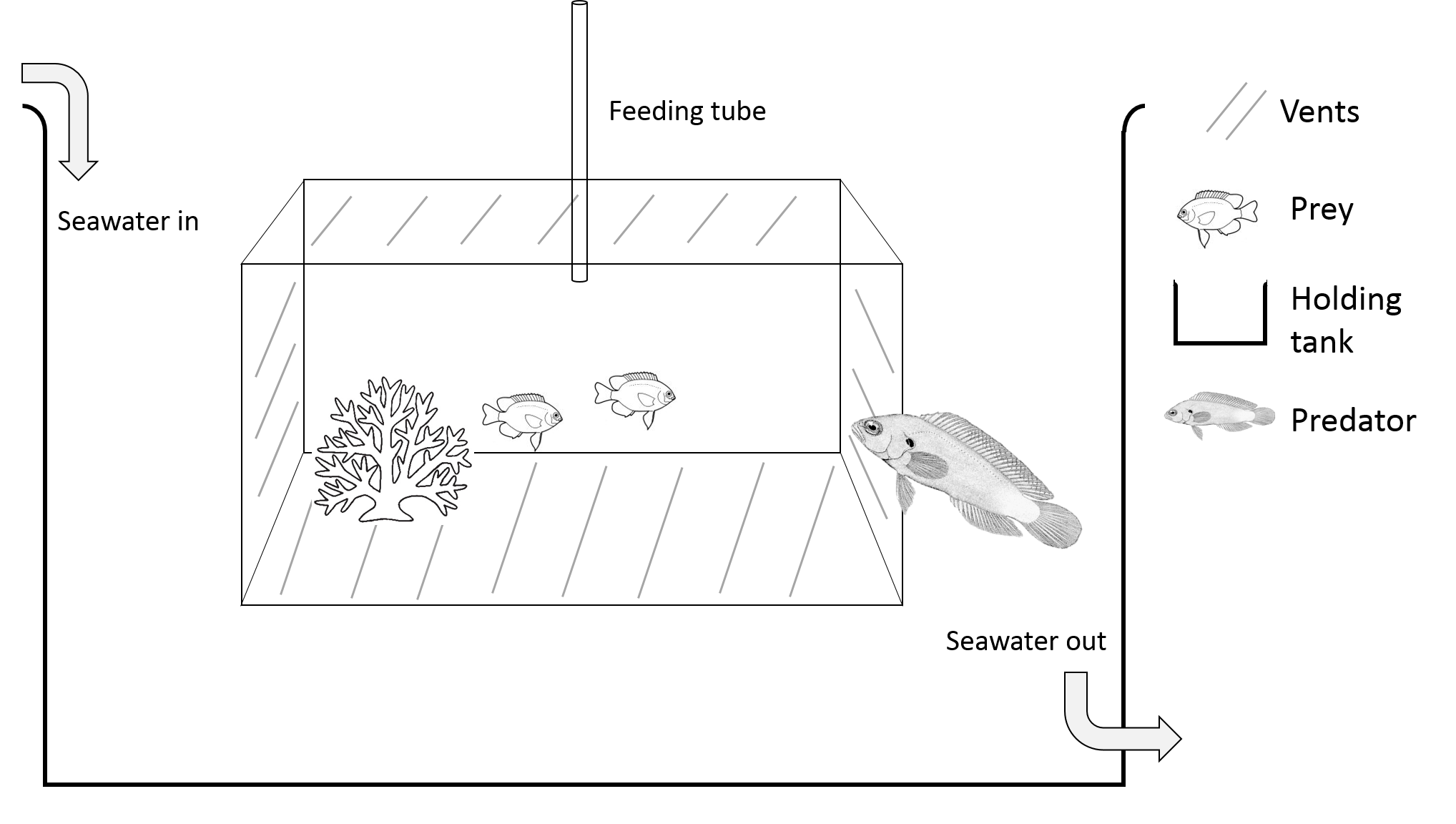

Supplement: S1 Fig — (TIF) [file pone.0151778.s001.tif]
